# Evaluation of the Young Deadly Free Peer Education Training Program: Early Results, Methodological Challenges, and Learnings for Future Evaluations

**DOI:** 10.3389/fpubh.2019.00074

**Published:** 2019-04-05

**Authors:** Belinda D'Costa, Roanna Lobo, Jessica Thomas, James Steven Ward

**Affiliations:** ^1^Sexual Health and Blood Borne Viruses Applied Research and Evaluation Network, Curtin University, Perth, WA, Australia; ^2^South Australian Health and Medical Research Institute, Adelaide, SA, Australia

**Keywords:** aboriginal, peer education, evaluation, methodology, sexual health promotion, blood borne viruses, sexually transmissible infections

## Abstract

Australian Aboriginal and Torres Strait Islander people experience disproportionately higher rates of sexually transmissible infections (STIs) and blood borne viruses (BBVs) when compared with the non-Indigenous population. Both incidence and prevalence data for bacterial STIs, such as chlamydia, gonorrhea, trichomonas, and syphilis in remote areas of Australia are reported at rates many times higher than that of non-Indigenous Australians. Similarly, rates of hepatitis B are disproportionately higher for non-Indigenous people in remote communities. The Young Deadly STI and BBV Free project was designed to increase the uptake of STI and BBV testing and treatment in young Aboriginal and Torres Strait Islander people living in remote and very remote areas of South Australia, Western Australia, Queensland, and the Northern Territory. Peer education formed one component of this pilot project and involved training up to 100 young Aboriginal and Torres Strait Islander people across 19 communities in a culturally appropriate and respectful manner on the transmission, testing, and treatment of STIs and BBVs. The trained peer educators were then required to deliver three community education sessions to young people in their respective communities in an effort to raise awareness about STIs and BBVs and encourage testing and treatment uptake. Preliminary evaluation findings, limited to the trained peer educators, revealed the peer educator training program contributed to STI and BBV knowledge gains among the trained peer educators and positively influenced their behavioral intentions and attitudes pertaining to STIs and BBVs. Working with remote Aboriginal and Torres Strait Islander populations on a highly sensitive, stigmatized topic presented many methodological challenges, particularly in terms of ensuring the collection of reliable evaluation data across geographically remote communities. The challenges and strengths associated with the implementation of the peer education training program along with implications for developing culturally inclusive evaluation practices will be discussed.

## Introduction

Sexually transmissible infections (STIs) and blood borne viruses (BBVs) are significant health issues within the Australian Aboriginal and Torres Strait Islander (hereafter Indigenous) population, with reports of disproportionately higher rates of STIs and BBVs among the Indigenous population when compared with the non-Indigenous population (1). Young Indigenous people and those in remote communities are particularly burdened by high rates of STIs and BBVs, with rates reportedly three to seven times higher than the rates experienced by non-Indigenous young people (1). Highlighting the scale of this issue, a recent review of the health and well-being of Australian Indigenous young people revealed STI rates among Indigenous 10–14 year olds were more than ten times the rate reported for their non-Indigenous counterparts (2). While reasons for this substantial disparity in STI rates are complex and varied—a manifestation of the ongoing impacts of colonization—factors such as limited and inconsistent contraceptive use, engaging in risky sexual behaviors due to alcohol and/or illicit drug use, and social disadvantage have been identified in the literature (3–5, 7).

In most instances, the more commonly reported STIs of chlamydia, gonorrhea, syphilis, and trichomonas are preventable, easy to diagnose, and successfully treated using inexpensive, readily available antibiotics (6, 8, 9). The diagnosis of STIs is often complicated, however, by the asymptomatic nature of these infections, causing infections to go unrecognized and untreated. With many Indigenous people typically seeking health care only in instances when illness symptoms present, reducing the prevalence of STIs through early diagnosis and treatment is particularly challenging (10). STIs that remain untreated can lead to adverse health outcomes, such as stillbirth, cardiovascular and neurological disease, infertility, death, and an increased risk of contracting human immunodeficiency virus (HIV) (6).

In addition to the limited distribution of health services in remote areas, service provision to young Indigenous people living in remote communities is further impeded by complex historical, cultural, and personal barriers, including (but not limited to) a mistrust of health services, culturally inappropriate/unwelcoming services, staff turnover, transport issues, financial costs, and in some instances a reluctance to access such services due to shame (3, 4, 8, 10, 11).

Evidently young Indigenous people in remote communities have a sustained personal vulnerability to STIs and BBVs, more so than their non-Indigenous peers (8). This suggests past approaches to remedy the situation have been unsuccessful. Sexual health education within the school context is an obvious and effective approach for improving sexual health outcomes for young people, however the often lower school attendance and retention rates of Indigenous young people compromise the ability of such programs to reach this population (8, 12). Thus, culturally appropriate, novel interventions that genuinely engage young Indigenous people and bridge the gap between clinical intervention and improved sexual health outcomes are urgently required for remote Indigenous communities throughout Australia. Peer education may provide the means for accessing this often hard-to-reach, yet high risk population on the culturally sensitive, highly stigmatized topic of sexual health.

Peer education is a well-established and internationally recognized approach to health promotion where information is shared between people of a similar age or from similar social groups (13). The prevailing assumption of peer-led approaches is that individuals are more inclined to personalize a message and change their behavior and/or attitude if the messenger is perceived to have similar characteristics or share similar concerns to oneself (14). While there is a dearth of literature reporting on the efficacy of peer education in addressing sexual health issues among young Indigenous people in the Australian context [e.g., (15)], a recent literature review of international Indigenous youth peer-led health promotion programs found some evidence to suggest that Indigenous peer health education is associated with positive outcomes (16). Specifically, among the studies reviewed, there were reports of improvements in knowledge, awareness, and attitudes, along with an increased use of health services and STI/BBV testing. Despite these positive findings, the evidence of behavior change was limited by the common methodological challenge of long follow-up periods.

### Program Overview

In 2016, the South Australian Health and Medical Research Institute (SAHMRI) was awarded Commonwealth funding to implement a youth peer education sexual health program in remote and very remote Indigenous communities throughout Australia in an effort to combat the disturbingly high rates of STIs and BBVs in these communities. Forming one component of the larger Young Deadly STI and BBV Free project (hereafter Young Deadly Free project), the peer education program involved training up to 100 young Indigenous people in 19 remote and very remote communities, across four jurisdictions, on the transmission, testing, and treatment of STIs and BBVs for the purpose of sharing such knowledge with, and positively impacting behavioral intentions and attitudes of, other young people in their respective communities.

### Young Deadly Free Peer Education Training Program

The development of the youth peer education training program was a collaborative endeavor. Key community stakeholders in remote health services were consulted on the program content to ensure topics were comprehensive and appropriate.

An audit was conducted by the SAHMRI project team to identify existing sexual health education and training programs shown to be age and culturally appropriate for young Indigenous people that could be adapted for the Young Deadly Free peer education program. Examples of resources identified included the condom and negotiation game developed by SHINESA (for more information, please see https://www.shinesa.org.au/product/condom-card-game-condom-negotiation-cards/) and the STI story developed by Sexual Health Quarters (WA) (17).

The Young Deadly Free peer education training program covered: anatomy and conception; STI and BBV transmission, testing, and harm reduction; and sex and the law. Group facilitation skills and practice in delivering content was also incorporated into the training program. Focus groups were run with young people to pilot the training program. The peer education training program was designed to be run over either a two or three day period, for approximately five to seven and half hours each day.

### Peer Educators

The program sought to recruit male and female peer educators who were well-respected community members with a passion for health and community. To be consistent with the demographics of the intended target audience for peer education, the peer educators had to be aged 16–29 years and identify as Aboriginal and/or Torres Strait Islander. Further to this, the eligibility criteria to train as a youth peer educator included: availability to run peer education sessions for other young people in their local community, English language literacy, sound communication skills and people skills, and non-judgmental attitudes toward sexual health, STIs, and BBVs. Recruitment of potential youth peer educators commenced first in Western Australia (WA) in September 2017. Some jurisdictions are still actively recruiting and training peer educators. At the time of writing, 46% (*n* = 46) of the anticipated 100 peer educators had been recruited and trained, comprising 25 females and 21 males aged 16–28 years, and median age 20 years. The peer educators are based in remote communities across three jurisdictions: WA (*n* = 27), the Northern Territory (NT) (*n* = 7), and South Australia (SA) (*n* = 11).

### Participating Communities

Nineteen remote communities, spanning three states and one territory, participated in the youth peer education program [refer to https://youngdeadlyfree.org.au/for-young-people/peer-education/ for map of communities]. A further nine communities were identified as potential program participants, but did not participate for varying reasons, such as failure to meet the eligibility criteria and offer to participate declined. The eligibility of communities selected to participate in the peer education program was assessed against the following criteria: a high prevalence of STIs and/or BBVs in the community; Accessibility/Remoteness Index of Australia (ARIA) classification of “remote” and “very remote” areas in SA, Qld, NT, or WA; a minimum population of 150 Aboriginal and/or Torres Strait Islander young people aged between 16 and 29 years; and an Aboriginal Community Controlled Health Service with membership of the peak state body, which utilizes electronic medical records that have the capability to extract STI/BBV testing data, or be willing to install data extraction software, and be willing to provide access to non-identifiable laboratory testing data. Following satisfaction of the eligibility criteria, community participation in the program required a formal agreement between the local health service and SAHMRI.

### Regional Coordinators

Six Regional Coordinators were recruited across the states and territory to ensure the implementation of the program at a jurisdictional level (WA, *n* = 2; SA, *n* = 1; NT, *n* = 2; Qld, *n* = 1). All Regional Coordinators had established community relationships and had extensive knowledge and experience in working with Indigenous communities in a range of capacities, such as nursing and health program implementation.

The Regional Coordinators attended a 3 day training session on how to deliver the peer education program to the prospective youth peer educators in an empowering educational environment. The training sessions were run by a trainer who was experienced in delivering health promotion and education to Indigenous youth in remote communities. The training adopted an experiential learning approach whereby the Regional Coordinators were actively involved in the training sessions, engaging in the games and activities that the youth peer educators would be running with the young people attending their sessions. This learning approach is said to improve one's ability to retain and execute the information being taught (18, 19).

Following the 3 day training session, Regional Coordinators commenced recruiting young people for the peer educator role. A range of recruitment strategies were used including posters, local Facebook groups, community newspapers, and radio. Some young people were nominated by their local Aboriginal health service, Elders, or other key stakeholders. Interested applicants were required to provide references from an Elder or another respected person in the community, such as a teacher. Those who met the eligibility criteria were supported to apply for a working with children check, including covering any costs incurred.

The Regional Coordinators delivered the peer education training program to small groups of young people (usually 2–8 people per group). Where possible, a male and female Regional Coordinator (or another project team member) worked in tandem to deliver gender specific training content, thus ensuring the gender norms traditionally associated with Indigenous culture were respected. Youth peer educators were each offered $100 at the completion of 15 h of peer educator training and a further $600 following the delivery of three community education sessions for other young people in their respective communities using content and activities from the peer education training program.

### Aims of Evaluation

Early in the program, SAHMRI commissioned the WA Sexual Health and Blood-borne Virus Applied Research and Evaluation Network (SiREN) to conduct an independent evaluation of components of the Young Deadly Free project, including the youth peer education program where the focus was on the training of the peer educators and the young people who attended the community education sessions conducted by the peer educators. This paper is limited to the trained peer educators, where the evaluation aimed to assess: (1) changes in knowledge, behavioral intention, and attitudes about STIs and BBVs among young Indigenous people training as peer educators; and (2) the suitability and impact of the peer education training program. The quality of peer education delivered and actual changes in behavior or changes in STI or BBV rates were outside the scope of the evaluation. Preliminary findings and methodological challenges associated with undertaking this large-scale, multi-site evaluation are discussed as are the key learnings emanating from this evaluation which may serve to inform future evaluations of similar nature.

## Materials (Design, Materials)

### Design

A mixed methods research design was used for the evaluation. This approach was favored to capitalize on the strengths of both quantitative and qualitative research approaches, including more culturally appropriate evaluation methods, and to accommodate lower literacy levels among some of the peer educators.

### Materials

This mixed methods evaluation utilized a knowledge survey and collected oral feedback from the peer educators. Supporting the data collection process was an evaluation guide developed for the Regional Coordinators.

### Knowledge Survey

The purpose of the self-completed knowledge survey was to assess knowledge, behavioral intentions, and attitudes about STIs and BBVs amongst the peer educators. The survey questions were extracted from larger surveys with demonstrated validity and reliability for this population cohort [for example, the Goanna Survey, see Ward et al. (20)]. The survey was paper-based and included 35 questions where respondents were generally required to select “yes,” “no,” or “don't know” answers. Two questions required respondents to indicate their level of agreement with each statement. The Regional Coordinators disseminated the surveys to the peer educators pre and post training (in most instances, immediately before and after training) to ascertain changes in their knowledge, behavioral intentions, and attitudes. For those peer educators with low English literacy, the Regional Coordinators provided assistance with the completion of the survey. The surveys remained anonymous, and did not include any identifying information to comply with the requests of some jurisdictions.

### Feedback Sessions

Following the training, oral feedback was collected from the peer educators to determine the suitability and impact of the youth peer educator training program. The Regional Coordinators, who had already established good rapport with the peer educators, conducted the feedback sessions by telephone or in person. The sessions occurred immediately after completion of the training to several days/weeks post training, depending largely on the availability of the Regional Coordinators and peer educators. The feedback sessions were conducted in an informal manner using a semi structured approach to ensure a degree of consistency across the participant sample, however, there was flexibility to ask other pertinent questions as they arose during the course of the session. An example of the types of questions asked included, “*How confident do you feel to run peer education sessions for other young people and why*,” and “*What was the most significant benefit for you attending the training?”*

Every effort was made to ensure the comfort and participation of the peer educators in the feedback sessions. Peer educators were given the option of providing feedback individually or in a group feedback session. They could also opt to provide feedback to their Regional Coordinator or to a Regional Coordinator from another region for the purposes of respecting cultural protocols related to gender norms or anonymity.

The feedback sessions ranged from 8–13 min (time required for informed consent additional) and were voice recorded and transcribed verbatim with participant consent. The Regional Coordinators also took written notes during the course of the feedback session to record observations and personal reflections.

### Evaluation Guide

An evaluation guide, specific to the youth peer education program, was developed by the evaluation team for the Regional Coordinators. The guide was designed to support the Regional Coordinators in the collection of evaluation data and included information on the evaluation tools, ethical considerations associated with data collection and storage, the transfer of data to the evaluation team, important evaluation dates, and the expectations of the Regional Coordinators in the evaluation. Included with the evaluation guide were labeled and prepaid envelopes to organize collected data and confidentially store and transfer such data to the evaluation team. Individual tele-meetings, up to 30 min in duration, were also held with each Regional Coordinator and a member of the evaluation team to discuss the evaluation in greater detail and address any specific questions.

## Results

The quantitative knowledge survey data were entered into SPSS (version 23) and frequencies were used to calculate summary descriptive statistics for the survey questions. Content analysis was used to analyze the data from the feedback sessions. This involved coding the transcripts to break down larger segments of transcribed data into smaller units to establish an understanding of its meaning. Common categories or themes were then identified.

### Quantitative Findings

The findings presented focus on the 6 month period from September 2017–March 2018. Forty six peer educators completed the pre knowledge survey and 40 completed the post knowledge survey. Responses were anonymous and could not be matched. The tables presented below highlight responses to the knowledge survey questions. Participant numbers vary marginally for some cells due to missing data. It is acknowledged that the differences reported between the pre and post groups may be accounted for by the six peer educators who did not complete a post knowledge survey.

### Knowledge Levels

The youth peer educators demonstrated a relatively high level of STI and BBV knowledge prior to commencing the peer education training (see Table 1). A high proportion of correct responses were provided for STIs in terms of symptoms, transmission, testing, and treatment and for BBV transmission, with the proportion of correct responses sitting at 56% or greater for 10 of the 13 questions. Participant knowledge was limited, however, for gonorrhea symptoms, trichomonas, and HIV curability.

**Table 1:** Participant responses to STI and BBV knowledge questions.

	**Pre knowledge survey** ***N*** **=** **46** ***n*** **(%)**			**Post knowledge survey** ***N*** **=** **40** ***n*** **(%)**		
	**Yes**	**No**	**Don't Know**	**Yes**	**No**	**Don't Know**
A man or woman can have a sex disease/STI without any obvious symptoms	32 (69.6)	3 (6.5)	11 (23.9)	36 (90.0)	1 (2.5)	3 (7.5)
Syphilis is a sex disease/STI that can harm a woman and her unborn baby	35 (76.1)	2 (4.3)	9 (19.6)	36 (90.0)	2 (5.0)	2 (5.0)
Chlamydia can be easily treated with antibiotics	30 (65.2)	0 (0)	16 (34.8)	34 (85.0)	2 (5.0)	4 (10.0)
Gonorrhea can be tested by a urine sample	28 (60.9)	4 (8.7)	14 (30.4)	36 (90.0)	1 (2.5)	1 (2.5)
Gonorrhea has no symptoms	19 (41.3)	9 (19.6)	18 (39.1)	29 (72.5)	4 (10.0)	7 (17.5)
Trichomonas is an infection that only affects women	7 (15.2)	8 (17.4)	30 (65.2)	14 (35.0)	18 (45.0)	8 (20.0)
The contraception pill or stick protects a woman from sex diseases/STIs	9 (19.6)	26 (56.5)	10 (21.7)	5 (12.5)	34 (85.0)	1 (2.5)
A HIV pregnant woman can infect her baby with HIV	32 (69.6)	1 (2.2)	11 (23.9)	36 (90.0)	2 (5.0)	2 (5.0)
There is a cure for HIV	8 (17.4)	19 (41.3)	18 (39.1)	7 (17.5)	32 (80.0)	1 (2.5)
Someone who looks healthy can pass on HIV infection	30 (65.2)	8 (17.4)	8 (17.4)	35 (87.5)	2 (5.0)	3 (7.5)
People who have injected drugs are at risk of hepatitis C	33 (71.7)	0 (0)	12 (26.1)	38 (95.0)	1 (2.5)	1 (2.5)
Hepatitis C can be passed on by tattooing and body piercing	34 (73.9)	1 (2.2)	11 (23.9)	38 (95.0)	0 (0)	1 (2.5)
Hepatitis B can be passed on through sex	28 (60.9)	4 (8.7)	14 (30.4)	31 (77.5)	5 (12.5)	3 (7.5)

Following peer education training there was a substantial increase in the proportion of correct responses for most STI and BBV knowledge questions, suggesting enhanced knowledge in these areas. Despite greater awareness of STIs and BBVs following the training, responses in the post knowledge surveys indicated continued uncertainty among the participants with regards to gonorrhea symptoms with a smaller proportion of participants correctly answering this question. Similarly, the proportion of correct responses to the trichomonas question remained <50% post survey, suggesting participant knowledge on this STI remained limited post training.

### Post Training Intentions

Generally, the participants indicated a high degree of intention in regards to discussions about STIs and BBVs as well as preventive practices (see Table 2 for all responses). Participant affirmative responses ranged from 70 to 83% in the pre knowledge survey for questions pertaining to talking about STIs, getting tested, and condom availability and usage. A steady increase in affirmative responses to these questions was observed in the post knowledge survey, where responses ranged from 80 to 95%, suggesting the peer education training was influential in encouraging participants to talk about STIs and BBVs and condom usage with future sexual partners. Of interest, <50% of the participants reported intending to get a STI or BBV test in the next 3 months (46% pre survey; 42% post survey).

**Table 2:** Participant responses to intention questions.

	**Pre knowledge survey** ***N*** **=** **46** ***n*** **(%)**			**Post knowledge survey** ***N*** **=** **40** ***n*** **(%)**		
	**Yes**	**No**	**Unsure**	**Yes**	**No**	**Unsure**
Do you intend to get a STI or BBV test in the next 3 months	21 (45.7)	12 (26.1)	12 (26.1)	17 (42.5)	14 (35.0)	8 (20.0)
Do you intend to have condoms with you if you have sex with a new partner in the next months?	38 (82.6)	4 (8.7)	3 (6.5)	36 (90.0)	3 (7.5)	1 (2.5)
Do you intend to talk about STIs if you have sex with a new partner in the next months?	32 (69.6)	5 (10.9)	8 (17.4)	32 (80.0)	2 (5.0)	5 (12.5)
Do you intend to talk about getting tested for STIs if you have sex with a new partner in the next months?	32 (69.6)	0 (0)	13 (28.3)	32 (80.0)	2 (5.0)	6 (15.0)
Do you intend to talk about using condoms if you have sex with a new partner in the next months?	38 (82.6)	1 (2.2)	6 (13.0)	38 (95.0)	2 (5.0)	0 (0)
Do you intend to use condoms if you have sex with a new partner in the next months?	35 (76.1)	1 (2.2)	9 (19.6)	38 (95.0)	2 (5.0)	0 (0)

### Attitudes to STI Testing

The pre knowledge survey showed high levels of agreement among the participants that STIs could seriously affect their health and that STI testing is a good thing (85% agreed, respectively). Similarly most participants agreed (78%) that they were confident they could get tested for STIs when they want, although there was concern among the participants regarding privacy and shame of STI testing with almost one-third agreeing that they were worried about the privacy/shame of STI testing. Many participants acknowledged their vulnerability to STIs, with 65% agreeing with the statement that they could easily get a STI. Responses in the post knowledge survey were relatively consistent for these STI statements, suggesting the peer educator training did not assist in alleviating concerns participants had about the privacy and shame associated with STI testing (see Table 3 for all responses).

**Table 3:** Participants' views about STIs and BBVs.

	**Pre knowledge survey** ***N*** **=** **46** ***n*** **(%)**			**Post knowledge survey** ***N*** **=** **40** ***n*** **(%)**		
	**Agree**	**Disagree**	**Neither**	**Agree**	**Disagree**	**Neither**
Getting a STI could seriously affect my health	39 (84.8)	1 (2.2)	3 (6.5)	34 (85.0)	1 (2.5)	3 (7.5)
I believe I could easily get a STI	30 (65.2)	6 (13)	7 (15.2)	26 (65.0)	6 (15.0)	6 (15.0)
I feel that I'm unlikely to get a STI	16 (34.8)	12 (26.1)	14 (30.4)	15 (37.5)	12 (30.0)	11 (27.5)
STI testing is a good thing	39 (84.7)	3 (6.5)	1 (2.2)	36 (90.0)	1 (2.5)	1 (2.5)
I'm confident I can get tested for STIs when I want to	36 (78.3)	2 (4.3)	4 (8.7)	34 (85.0)	1 (2.5)	3 (7.5)
I'm worried about the privacy and/or shame of STI testing	15 (32.6)	20 (43.4)	9 (19.6)	11 (27.5)	17 (42.5)	10 (25.0)
I don't know when I should get a BBV test	26 (56.5)	6 (13.0)	10 (21.7)	12 (30.0)	21 (52.5)	5 (12.5)
Young people should get a BBV test if they've been in prison	31 (67.4)	0 (0)	11 (23.9)	33 (82.5)	2 (5.0)	3 (7.5)
Young people should get a BBV test if they've had sex without a condom	37 (80.4)	0 (0)	6 (13.0)	32 (80.0)	2 (5.0)	4 (10.0)
Young people should get a BBV test if they've shared injecting equipment	39 (84.8)	0 (0)	3 (6.5)	37 (92.5)	1 (2.5)	0 (0)

### Attitudes to Testing for Blood Borne Viruses

In relation to participants' views about BBVs, there was limited knowledge among the participants in regards to BBV testing with just over half of the participant sample (56%) agreeing in the pre survey that they do not know when to get a BBV test, decreasing to 30% in the post survey. Despite the uncertainty demonstrated in the pre knowledge survey, there was relatively high agreement among the peer educators about the types of situations that warrant BBV testing, with 67% agreeing that a BBV test is needed if one has been prison (increasing to 82% post survey), 80% agreeing that a BBV test is needed if one has had sex without a condom (remaining consistent at 80% post survey), and 85% agreeing that one should get a BBV test if injecting equipment has been shared (increasing to 92% post survey). Collectively the post knowledge survey responses suggest the peer education training increased knowledge about BBV testing among the participants.

### Qualitative Findings

Feedback sessions were held with nine peer educators (5 males, 4 females) in SA and WA. One group feedback session was held with five male participants, and four one-on-one feedback sessions were conducted with female participants. Two feedback sessions took place in person, three were conducted via telephone. The feedback sessions were concise in length and as such, data were limited and may not represent the views and experiences of all trained peer educators. Responses to the feedback questions have been analyzed and are presented below.

### Training Experience

The participants reflected positively on their peer educator training experience, reporting that the informative and interactive nature of the training made for an enjoyable training experience. When asked to rate the training overall on a scale of 1–10, where 1 denoted “very poor” and 10 denoted “excellent,” the participants offered a mean score of 7.7 (mode = 8). Specifically the participants were particularly praising of how the information was presented, the cultural appropriateness and sensitivity of the information, the location of the training, and the effectiveness of the facilitator with each of these receiving mean scores ranging from 9.2 to 9.7.

Participants commented on the importance of respecting cultural norms by offering gender specific training as indicated by the following remark by a male participant, “*I think the fact that it's gender specific for us has worked extremely well*,” while another female participant remarked, “*When we do the teachings, we have to like avoid, like you know, females go with the females…we won't like educate the men, like it would be inappropriate if we did that.”*

The relevance and usefulness of the content and the payment received for attending the training were also rated highly, with mean scores of 8.7 and 8.3, respectively. Receiving slightly lower ratings were the length of the training (mean = 7.8) and the behavior and participation of the other participants (mean = 7.4), with one female participant reporting that the topic caused shyness among some of the peer educators because “*they don't like talking about them sort of things.”*

### Confidence in Running a Community Education Session

Six of the nine participants (male and female) reported feeling either “quite confident” or “very confident” in running a community education session with other young people. Peer educators reported various factors contributed to feelings of confidence including: knowledge acquired from training or related work experience; having the support of other peer educators; and knowing the young people who would be participating in the community education sessions. Examples of the feedback provided by the peer educators (male and female) included, “*I feel more confident purely because of the information now, I wouldn't be guessing now, I actually have got some info that I can go to now”* and “*Because I feel like I've learnt like a lot and like now that I know I'm like more confident in telling people.”*

For the three male participants who reported a lack of confidence in running a community education session with young people, concern was generally related to a fear of public speaking, “*Probably cos I not a person to be talking in front of people”* and embarrassment discussing the subject matter with others “*I can do it, but sometimes I feel a bit shame.”* Some of the participants who reported low confidence levels in the delivery of community education sessions indicated that they would feel comfortable speaking with other young people in a one-on-one context or if they had someone co-present with them.

### Value of Community Education Sessions for Young People

When asked to report on how valuable the community education sessions will be for young people in the community, the participants were of similar opinions that the sessions would be either “quite valuable” or “very valuable”—the highest rating among the four possible options. Responses from the male and female participants included, “*A lot of people don't, aren't educated in this specific area and we just all assume everyone knows what to do…in that area”* and “*If they're delivered effectively, it could be extremely valuable.”*

### Suggested Improvements for the Training

Participants did not identify many improvements to the peer education training, however some participants acknowledged that shorter training days, possibly spread over several days, would be a helpful improvement. A female participant noted that the training could be improved by “*Going to a place where people don't go”* suggesting a private setting for the training is an important consideration.

### Benefits of Attending Training

The participants were unanimous in their opinions that the most significant benefit they gained from the peer educator training was the knowledge they acquired. One male participant reported, “*Hearing about all types of diseases that you didn't even think of, wouldn't even think about.”* A female participant remarked, “*The best thing was the, some stuff you told me about sexually transmitted disease, that I didn't know of, now I know.”* This knowledge acquisition was said to be empowering for the participants, with one female participant stating, “*It's just like empowered us to just, you know, start educating our family members, trying to avoid early pregnancies, and you know, STIs and stuff.”*

## Discussion

Seeking to remedy the alarmingly high rates of STIs and BBVs among young Indigenous people, the Young Deadly Free project implemented a youth peer educator sexual health training program in 19 remote and very remote communities. Peer education has had limited application in sexual health promotion in the Indigenous Australian population context, despite increasing evidence attesting to its effectiveness (15, 21–24). Using a knowledge survey and feedback sessions with young people who trained as peer educators, the preliminary evaluation findings from the peer educator training program suggest the training contributed to knowledge gains in STI and BBV transmission, testing, and treatment among the Indigenous youth peer educators and positively influenced behavioral intentions and attitudes in this cohort.

Specifically, the pre knowledge survey revealed relatively high levels of STI and BBV knowledge, and high levels of agreement in relation to behavioral intentions and attitudes to STI and BBV testing among the peer educators prior to engaging in the peer educator training. This may be due in part to the project seeking to recruit trainee peer educators with an interest in health and community issues and as such, the peer educators were knowledgeable on the topic. Despite the pre-existing high levels of STI and BBV knowledge among the peer educators, a steady increase in knowledge levels was observed following the training—this was particularly apparent for questions pertaining to HIV curability and BBV testing. Limited knowledge about gonorrhea symptoms and trichomonas remained among the participants post training, however, and some participants continued to report concerns about the privacy and shame of STI testing, suggesting more of a focus on these topics is warranted in the peer educator training.

Australian research by Mikhailovich and Arabena (15) also reported improved knowledge among Indigenous peer educators who had participated in a youth peer education training program on sexual and reproductive health. While research involving other youth peer educators across varied contexts have revealed acquired knowledge as an important outcome of their peer education training, with some also reporting a change in their views and attitudes following the training (25–28).

The current peer educator training also enhanced feelings of confidence among the trained peer educators, with more than half of the peer educators who provided oral feedback reporting feeling quite confident or very confident in conducting community education sessions with other young people in their community. Additional support from the Regional Coordinators may assist those peer educators who reported a lack of confidence following the training, particularly in terms of minimizing concerns associated with public speaking and feelings of shame and/or embarrassment when speaking about sexual health with peers. Encouragingly, the trained educators reported that the community education sessions would be valuable for young people in their respective communities due to the lack of knowledge that exists on STIs and BBVs.

The favorable outcomes associated with the peer educator training could be attributed to the format and content of the training, which was highly regarded by the participants as being culturally appropriate and respectful, and also of relevance to the target audience. This is consistent with other studies highlighting the importance of adhering to cultural protocols in the delivery of STI and BBV information when seeking to engage young Indigenous people on such a culturally sensitive topic [e.g., (29)]. It is expected that the hands-on, experiential style of the peer educator training will benefit the trained peer educators when sharing information about sexual health with peers in their community, both formally through the community education sessions and via informal conversations with peers, family, and the wider community.

### Challenges

The positive outcomes associated with the peer educator training is noteworthy, particularly when considering the context in which the program and evaluation exist. Most notably, the program was implemented in remote communities, many of which are either experiencing higher priority issues that supersede the importance of sexual health, or are feeling disillusioned by research or suffering research fatigue (30). Thus, encouraging the uptake of the program in communities such as these requires significant time and the development of trust and rapport with key people in the community—considerations that are often incompatible with the tight timeframes imposed by external funding bodies.

Further to the above are the methodological challenges associated with undertaking an evaluation of a multi-jurisdictional program in remote Indigenous communities, commencing with the need to obtain ethics approval from multiple ethics committees for the conduct of the research. This was a time intensive process, due in part to the complexity of the larger project for which ethics approval was sought, different requirements and processes of each committee, including the frequency with which some of the committees met. Future programs of a similar nature will benefit from allocating sufficient time to the ethics approval process.

The multi-jurisdictional nature of the program, and the geographical spread of the participating sites, also presented challenges for data collection, which necessitated a heavy reliance on the Regional Coordinators to collect and transfer evaluation data to the evaluation team based in WA. With great variation in the evaluation skill set and experience among the Regional Coordinators, processes had to be implemented to maximize the reliability and validity of the data collected, as well as the secure transfer of data to the evaluation team. The development of the evaluation guide for the Regional Coordinators as well as the supply of labeled and pre-paid envelopes for the organization and return of data are examples of how the evaluation team sought to manage the collection and transfer of data across the 19 remote sites. While one may question the methodological rigor of the data collection responsibility residing with the Regional Coordinators, it was important not only from a logistical perspective but also from an ethical perspective, for it helped to ensure adherence to culturally secure evaluation methods and provided the opportunity for data to be collected from different sites in parallel rather than serially by the evaluation team.

### Strengths

The methodological challenges faced in the evaluation of the peer educator training program are evidence of the complexities associated with evaluating a multi-jurisdictional program based in remote Indigenous communities. Notwithstanding these challenges, there are indeed strengths of the evaluation design that have helped to mitigate some of these challenges that are worthy of discussion. Communication has been key to managing many of the methodological challenges that have presented in this evaluation. Undertaking a multi-jurisdictional evaluation where the evaluation team are not active in the data collection process requires the establishment of effective communication between the evaluators and regions. This was established through an initial face-to-face meeting and then via regular email and telephone contact where training and support in the collection and handling of data was provided, and the opportunity to debrief was offered. The development of an evaluation guide for the Regional Coordinators also aided the data collection process and ensured consistency across the program sites, while the data transfer process was efficient to maximize the return of completed and organized data to the evaluation team.

The experience of the evaluation team and the flexibility of the team over the extended project timeframe are additional strengths of the evaluation design. Furthermore, the involvement of the evaluation team in the design of the peer education program is a notable strength for this permitted the team to have input into the design of the data collection tools and methods of data collection. It is highly recommended that future projects with an evaluation component seek to engage the evaluation team early in the project design.

The strengths of the peer education program governance must also be acknowledged for contributing to the success of the program. To begin, the establishment of a central program coordinator role assisted with minimizing the impact of geographical distance in a program of this scale. This role was responsible for the management of the program at a national level and facilitated the implementation of the program and evaluation. The recruitment of Regional Coordinators who were committed and well-engaged in their respective communities also served to benefit the program and helped to ensure its implementation at a jurisdictional level.

The program's strengths-based approach is another recognized quality. At the core of the program is the objective to upskill and build the capacity of young people in the community to make informed decisions about their health and body. The program works collaboratively with local Aboriginal Medical Services, Elders, and other people of influence to encourage participation by young people. This type of approach can energize a community, particularly those that are, or have been, facing challenging issues.

## Limitations of the Evaluation

The cultural protocols and sensitivities surrounding not only the topic, but the conduct of research in Indigenous communities, combined with the geographical spread of the multiple remote program sites have presented challenges that have required careful consideration. The evaluation was not, however, without limitations, and following are ways of improving evaluations of this nature. Initially a detailed environmental scan of the proposed participating communities is recommended to determine the community's readiness for research and to ensure they have the capacity and interest to commit and support the proposed research. The establishment of realistic timeframes is also of paramount importance when undertaking research in remote Indigenous communities. Such timeframes must be flexible to changes in the research process and also take into consideration the time required to establish trusting relationships in Indigenous communities. The importance of time and flexibility when working with Indigenous communities has been noted elsewhere [e.g., (30)]. Similarly, extended projects such as this require strategies for maintaining momentum and consultation and an acknowledgment that key contacts and signatories in communities may change by the time the project is implemented.

In instances where evaluators do not collect data and outsource this responsibility to other personnel, it would be beneficial to establish a baseline of the evaluation and research skill set among those collecting the data in order to provide more comprehensive, targeted support to those less experienced. Similarly, allocating more resources to the data collection process, such as having an evaluator observe a training session, would be advantageous to improve the reliability and consistency of the data being collected. Also, greater consideration should be afforded to the data collection tools and the rigorous testing of such tools with the target population. In the present evaluation, the anonymous nature of the knowledge surveys placed limitations on data analysis, only permitting comparisons of the pre and post survey data in terms of proportions. Independent *t*-tests to establish the significance of changes observed between the pre and post knowledge surveys were not possible. Future evaluations of this type could consider how one assures participants of the anonymity of their survey responses, while also allowing for a higher level of analysis of survey data.

Lastly, the evaluation findings cannot determine the ability of the peer educators to go on to deliver effective, factually accurate community education sessions with other young people. The evaluation also did not assess whether some peer educators were more effective than others, in what ways, or the reasons which may have contributed to this (e.g., age, gender, or differences among the Regional Coordinators in terms of training and data collection). Nor was it possible to determine whether the reported gains in STI and BBV knowledge along with the reported attitudinal changes, will translate into safer sexual practices and ultimately a reduced prevalence of STIs and BBVs among this population cohort. Such factors are important in determining the effectiveness of peer education in sexual health promotion and in influencing changes in behaviors including safer sex practices, testing and treatment of STIs and BBVs. While the full impact of peer education will not be assessed by the methods used for this evaluation, the indirect effects of the peer education program may be observed through increased access to health services and reduced STI and BBV rates in the communities. The final evaluation of this program may provide the opportunity to assess the extent to which the peer education program has positively impacted on the various remote communities.

## Conclusion

The preliminary findings from the evaluation of the Young Deadly Free youth peer educator training program demonstrate the training program contributed to knowledge gains in STIs and BBVs among the young people training as peer educators and positively influenced behavioral intentions and attitudes in this cohort. These findings are promising for the implementation of the community education sessions in the participating sites, which will provide evidence attesting to the efficacy of the peer education model for sexual health promotion within this population. Determining the long term impacts of the peer education program is a worthy line of enquiry and subsequently will form an important focus of the larger evaluation of the Young Deadly Free project.

Undertaking evaluation work in research fatigued populations on a topic abound with cultural sensitivities presents a myriad of challenges, however, this should not dissuade one from engaging in such important work. It requires a departure from traditional evaluation methods where the evaluators must work within the confines of the requests of the ethical committees and the preferences of the participating communities while also balancing the need to collect valid and rigorous data. The identified strengths of the Young Deadly Free youth peer educator training program evaluation, along with the reported limitations, make valuable contributions to the limited evidence base for best practice evaluation in remote Indigenous communities.

## Ethics Statement

This study was carried out in accordance with the recommendations of Values and Ethics: Guidelines for Ethical Conduct in Aboriginal and Torres Strait Islander Health Research (2003) and the National Statement on Ethical Conduct in Human Research (2007) with written informed consent from all subjects. All subjects gave written informed consent in accordance with the Declaration of Helsinki. The protocol was approved by the Curtin University Human Research Ethics Committee; Western Australian Aboriginal Health Ethics Committee; Human Research Ethics Committee of the Northern Territory Department of Health and Menzies School of Health Research; Central Australian Human Research Ethics Committee; and the Aboriginal Health Research Ethics Committee of the Aboriginal Health Council of South Australia.

## Author Contributions

RL and BD contributed conception and format of manuscript. BD performed quantitative and qualitative data analysis, and completed manuscript drafts and revisions. JT wrote a section of the manuscript. RL critically reviewed manuscript. JW is the principal investigator on the Young Deadly Free project and approved the publication of content pertaining to evaluation activities. All authors had the opportunity to comment and approve the version submitted.

### Conflict of Interest Statement

The authors declare that the research was conducted in the absence of any commercial or financial relationships that could be construed as a potential conflict of interest.
